# Mechanisms Influencing Maximum Joint Range of Motion and Stiffness: A Narrative Review

**DOI:** 10.1007/s40279-026-02491-8

**Published:** 2026-08-21

**Authors:** Camila D. Lima, Anthony J. Blazevich

**Affiliations:** 1 Institute of Physics, Gleb Wataghin, University of Campinas, Campinas, Brazil; 2 Centre for Precision Health, School of Medical and Health Sciences, Edith Cowan University, 270 Joondalup Drive, Joondalup, WA 6027 Australia

## Abstract

Joint ‘flexibility’ is an encompassing term influenced by both a joint’s maximum range of motion (ROM_max_) and its resistance to rotation, i.e. its stiffness. A critical amount of flexibility is thought to be important for both sports performance and daily and occupational tasks. Improvements in joint ROM_max_, specifically, are often assumed to result from material and mechanical changes in the joint’s soft tissue structures; however, they also may result from changes in sensory information relayed from peripheral receptors to the brain, or the interpretation of that information. Changes in psychological state mediated by pain, state arousal and other psychological factors can therefore influence the voluntary capacity of individuals to tolerate a stretch, and thus their ROM_max_. Stiffness, on the other hand, may be affected by both muscular and non-muscular tissues, although the most important sources of stiffness are yet to be agreed upon. In this narrative review, we define important terms as well as the factors impacting stiffness and ROM_max_. We assert that joint stiffness is affected by the extracellular matrix, intramuscular fat and water as well as fascicle rotation, and that these factors also by extension may affect ROM_max_. While sarcomere (and fibre) stiffness is largely regulated by the giant protein titin, it plays a lesser role at the whole muscle level. Alternatively, ROM_max_ can be influenced by our processing of sensory information and the decisions we make (consciously or subconsciously) about our willingness to stretch further. Thus, cognitive and attention processes can influence an individual’s decision to stop a stretch.

## Key Points

**Table:** 

Whole muscle passive stiffness is predominantly regulated by the extracellular matrix (ECM), including its composition, architecture and capacity to remodel, with potential additional contributions from fascicle rotation capacity, intramuscular fat and water content; in contrast, titin’s role appears largely confined to the sarcomere level, and tendon stiffness does not appear to be a key determinant of either stiffness or maximum range of motion (ROM_max_).
ROM_max_ is only partly determined by tissue mechanical properties; sensory–perceptual and cognitive processes, including the interpretation of proprioceptive signals from muscle spindles, nociceptors and joint mechanoreceptors within higher brain centres, critically shape an individual’s voluntary stretch tolerance and thus their ROM_max_.
A dual-mechanism model is proposed in which passive stiffness is principally a tissue-level phenomenon whereas ROM_max_ additionally reflects neuro-perceptual decision-making, suggesting that interventions targeting cognition and pain perception may complement mechanical approaches such as stretching or eccentric training for improving joint flexibility.

## Introduction

Joint (i.e. muscle) flexibility can be defined as the maximum joint range of motion (ROM_max_) or the resistance to joint rotation under a specific set of conditions [14]. The strength of sensory feedback to the central nervous system, which coordinates reflexive sensory and descending (volitional) activity as well as the resistance to stretch of the peripheral soft tissue network, including articular capsules, ligaments, muscles and tendons (and the whole muscle–tendon unit), combine to influence flexibility, at least when bony structures do not play a role (elbow extension, for example, is limited by ulno-humeral contact). Thus, both the resistance to stretch, i.e. stiffness, and ROM_max_ may be affected by neuromuscular differences between individuals or changes over time, including differences in muscle–tendon unit (MTU) mechanical properties, registration of stretch signals, transmission of signals via Ia afferents to the spinal motoneuron and cerebellum, and transmission of pain, joint angle, pressure or other information back to the central nervous system (CNS) [78]. Because many of these processes can be regulated by descending cortical connections, factors such as psychological state (including level of arousal) or sensitivity to pain might influence an individual’s voluntary decision to stop stretching further, and thus also influence measured flexibility. However, little is currently known about the relative importance of physical versus psychological factors and whether this varies with conditions (stretch speed, level of motivation or stress, voluntary activity during stretch) or across joints (muscle groups).

Understanding the factors that influence both stiffness (resistance to stretch) and ROM_max_ is an important first step in developing interventions to improve joint flexibility, and thus mobility, functional independence [12, 110, 111], and both occupational and sporting movement capacities [9, 51]. The aim of the present review is to explore the neuromuscular factors that might influence both stiffness and ROM_max_ under varying conditions (e.g. speeds, forces, etc.) of muscle stretch, which may then prompt the future identification of new interventions and safe exercise prescriptions in clinical and sports conditions. This review focuses primarily on understanding these factors within healthy populations. In doing so, we assess whether the available evidence is consistent with a model in which stiffness and ROM_max_ are influenced by related but partially distinct mechanisms.

## Search Strategy and Review Approach

This article is a narrative review, an approach selected because the aim of synthesising mechanistic evidence across multiple biological levels, from molecular sarcomere properties to cortical decision-making, requires the integration of heterogeneous study designs and animal and human models that are not amenable to the quantitative synthesis of a systematic review or meta-analysis. The review was conducted in accordance with the Scale for the Assessment of Narrative Review Articles (SANRA) reporting recommendations [6]. Systematic searches were conducted between 6 July 2020, and 4 May 2026, by two independent researchers across six electronic databases: PubMed, SPORTDiscus, Google Scholar, a university library database, Web of Science, and Medline using combinations of search terms including joint, muscle, or tendon with tightness, stiffness, elasticity, ROM, or flexibility. Two independent researchers screened the titles and abstracts of all retrieved articles; conference abstracts were excluded. Where primary studies were insufficient to explain an underlying biomechanical or physiological mechanism, additional studies were incorporated to provide the necessary mechanistic context.

## Soft Tissue Contributions to Stiffness and Joint Maximum Range of Motion

**Fig. 1:**
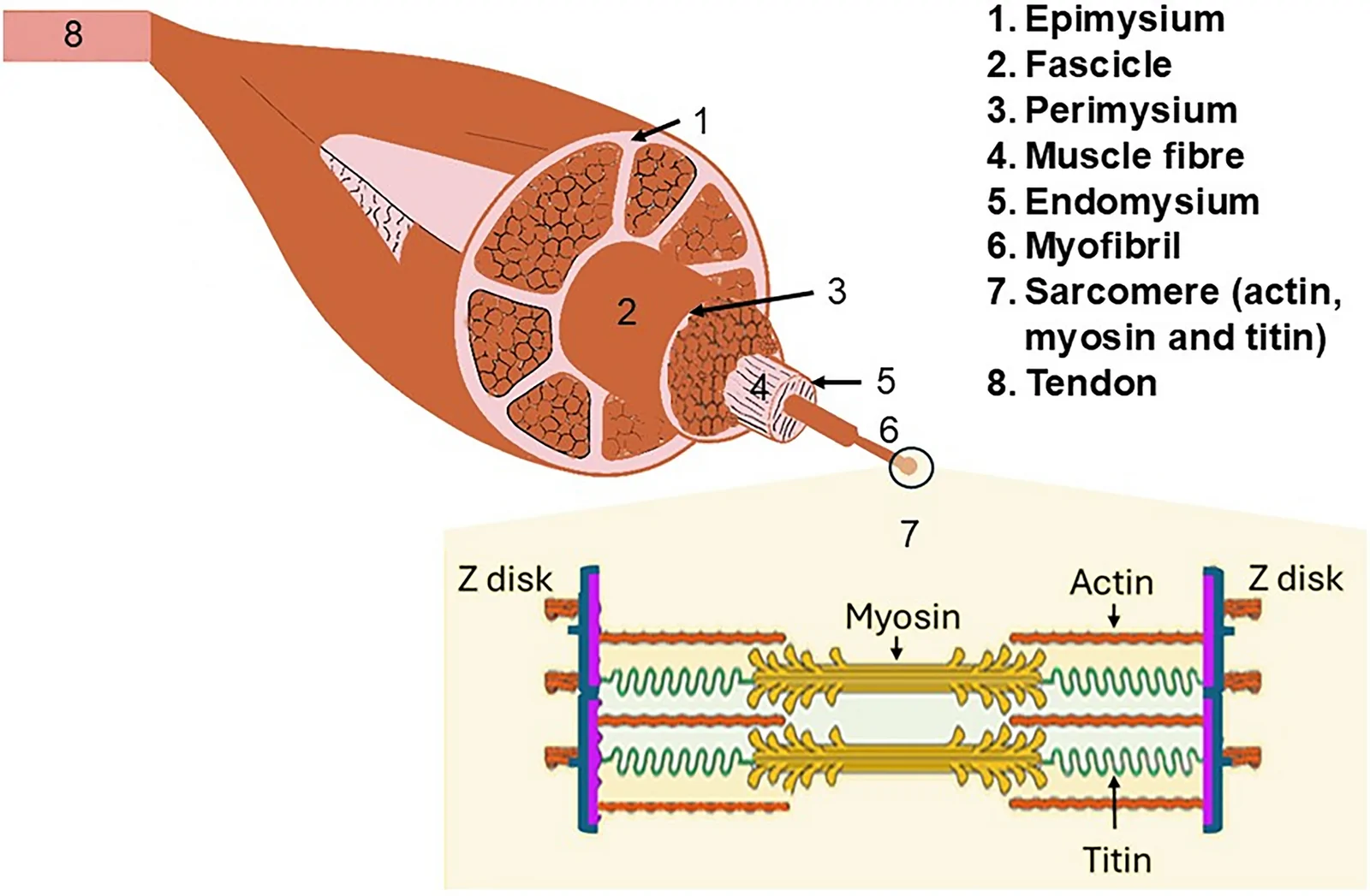
Skeletal muscle macro- and microscopic structure

During an unconstrained task, stiffness can be strongly affected by the mechanical properties of the soft tissues, including the muscles, which are largely composed of collagen protein (~ 80% by dry mass for connective tissues) and water [11]. Soft tissues (muscle, tendon, extracellular matrix [ECM]) are collagen rich, with a coordinated and relatively consistent arrangement [72]. Skeletal (striated) muscle has a ‘hierarchical’ structure, with muscle fibres sheathed by an endomysium, bundles of fibres (fascicles) surrounded by a perimysium, and the whole muscle overlaid by the epimysium and interdigitated by fascia (Fig. 1). These largely collagenous networks contain other substances such as water, other proteins and polysaccharides to form a complex ECM, which surrounds many body structures and strongly influences their function [84]. The ECM also surrounds the tendon, which links the muscle to its adjacent bones to form the muscle–tendon unit. The tendon is a strong yet somewhat compliant tissue, also primarily composed of collagens, and is continuous with its ECM.

Many different types of collagens exist, with their fibrillar structure (longitudinal arrangement) allowing high tensile forces to be effectively transmitted between tissue components [56, 122]. Collagens, and the structures formed by them, also have viscoelastic properties, ensuring that their mechanical properties are affected by the rate at which they are deformed and the time over which they encounter external forces. Thus, their properties, and the properties of the tissues they constitute, vary with changes in movement speed and loading duration. Indeed, non-collagenous MTU components also display viscoelastic behaviour and may impact tissue stiffness differently during movements at different speeds and of different durations. Since movement speed in particular is influenced by the rate of MTU stretch and shortening, it should strongly influence ROM_max_ and stiffness [76, 97]. Viscous behaviour is affected by extracellular and intracellular fluid pressures, which then influence movement within the muscle (and tendon) ECM [73], which should then influence the muscle’s resistance to stretch since intramuscular connective tissues are critical to muscle stretch properties [85], as described in detail below. Viscous behaviour therefore affects MTU stiffness in a rate-dependent manner, measured in vivo as the slope of the torque–angle relation [38, 97], and this should be remembered when assessing the research described below, during which data are predominantly captured during slow-speed muscle–tendon stretches. (Fig. 1 and Table 1).

**Table 1:** Each check mark (✓) represents the estimated likelihood of contribution between factors

	Stiffness	Maximum range of motion (ROM_max_)	Model	Joint
*Structural level*				
Whole muscle	✓✓✓	✓✓	Animal and human	Hips, knee, ankle and elbow
Extracellular matrix	✓✓✓	✓	Animal and human	Hips, knee and ankle
Epimysium	✓✓	?	Animal and human	Hips and ankle
Perimysium	✓✓✓	?	Animal	N/A
Endomysium	✓	×	Animal	N/A
Fascicle	✓✓	✓		
Lengthening (during stretch)	✓	?	Animal and human	Hips, knee and ankle
Rotation (during stretch)	✓	?	Animal	Hips, knee and ankle
Fibre	✓	✓	Animal and human	Hips, knee, ankle and elbow
Sarcomere number	✓	?	Animal and human	Hips, knee and ankle
Fibre stiffness	✓	?	Animal and human	Hips, knee and ankle
Sarcomere	✓	×	Animal and human	Hips, knee and ankle
Actin myosin cross-bridges	×	×	Animal	N/A
Titin	✓	×	Animal	N/A
Tendon	✓	✓	Animal and human	Knee and ankle
Intramuscular fat	✓✓✓	✓✓	Human	Hips, knee and ankle
Intramuscular water	✓✓✓	✓✓	Animal and human	Hips, knee and ankle
Joint capsule, ligaments, and articular connective tissue	✓	✓✓	Human	Elbow
*Neurophysiological level*				
Sensory information	✓	✓✓	Human	Ankle and elbow
Pain receptors	✓	✓✓	Human	Ankle and elbow
Muscle spindles	✓	✓✓	Animal and human	Ankle and elbow
Joint receptors	✓	✓✓	Animal and human	Ankle and elbow
Interpretation of sensory information (brain)	?	✓✓	Human	Knee, ankle and wrist

✓✓✓ Strong evidence: demonstrated across independent studies with experimental models (human and animal) and different joints

✓✓ Moderate-to-strong evidence: consistent findings in high-quality studies, but evidence may be primarily limited to a specific model or a single joint

✓ Poor-to-moderate evidence: limited data, although generally confirmatory of a strong hypothesis

(?) Unclear/emerging evidence: conflicting results exist in the literature, or theoretical importance is high but empirical data is currently insufficient

(x) Limited evidence of contribution or no effect: initial studies suggest a negligible or very minor role in MTU mechanics under physiological conditions

### Sarcomere-Level Contributions

Muscle fibres consist of a parallel arrangement of myofibrils, which are in turn formed by the serial arrangement of sarcomeres. Each sarcomere contains actin proteins anchored directly to the Z disk and myosin proteins held in place by the giant protein titin, which functions as a spring and is also anchored to the Z disk [105]. Previous studies have indicated that the stiffness of active cross-bridges as well as the actin and myosin filaments themselves make a relatively small contribution to whole muscle stiffness [71]. In fact, in an inactive sarcomere, relatively few structures are found to bear load during elongation, these being the Z band, titin, and any weakly bound cross-bridges [71]. Given that the Z band is exceedingly narrow compared with the overall length of the sarcomere, any expansion is likely to have minimal effect on overall sarcomere lengthening [43]. In any case, the Z band appears impervious to deformation during passive sarcomere stretch despite it deforming by ~ 20% during active muscle contraction [43]. According to these results, even in muscles operating on the plateau or the descending limb of the sarcomere-length relation [71], the contribution of cross-bridges to passive stiffness is usually small, and therefore stiffness of the sarcomere itself largely arises from the deformation of titin [7]. Indeed, titin is especially important when stretches occur under different rates and amplitudes [7] as described in more detail in the following section. (Fig. 1 and Table 1).

#### Titin Contribution to Sarcomere Stiffness

Titin is the massive protein linking the myosin molecule to the Z band consisting of a myoglobulin domain and fibronectin. Within its complex structure, the I band part of the molecule contains an Ig domain (serially linked immunoglobulin-like domains) and a PEVK segment [rich in proline (P), glutamate (E) valine (V), and lysine (K)] [105]. The PEVK segment is the main extensible segment in the molecular spring region of titin and resides in the part of titin that is not directly bound to myosin [21]. Removal of a group of exons from the titin gene in mice led to a significant (~ 75%) reduction in PEVK segment length and a marked increase in fibre and whole muscle passive stiffness, indicating that titin can affect passive fibre stiffness in skeletal muscles [21], at least when its properties are changed substantially. Furthermore, overall titin compliance has been assessed by mechanically and chemically isolating myofibrils and utilising silicon nitride levers to assess force levels [56, 57, 70]. Using this method, Leonard and Herzog (70) blocked actin and myosin interaction and showed that a structure other than actin or myosin must be responsible for the increased tension in passively stretched myofibrils whilst titin elimination reduced passive resistive forces to near zero. Such results directly implicate titin as a strong contributor to passive myofibril (and thus fibre) stiffness. Nonetheless, although the titin contribution to myofibril and fibre stiffness appears to be significant, these experiments do not provide conclusive evidence of its influence on whole muscle stiffness, as described in the following sections.

#### Effect of Serial Sarcomere Number

Muscle fibres are composed of myofibrils that consist of series of sarcomeres (Fig. 1), so individual sarcomere stiffness and the number of sarcomeres in series should cooperatively affect fibre, and possibly whole muscle, stiffness [36]. Since the stiffness of a material or structure is inversely proportional to its length, longer myofibrils or fibres should theoretically be more compliant than shorter myofibrils and fibres, i.e. stiffness should be inversely proportional to the number of serially arranged sarcomeres in each myofibril or fibre. It is thus accepted that long muscles (with long fibres) are more extensible than short muscles [15, 19, 122].

Several studies have induced sarcomere number adaptation by immobilising animal muscles at short or long lengths for several weeks [17, 85, 121, 127, 128]. In these studies, decreases in serial sarcomere number have been observed in rat [54], mouse [129] cat [121], and rabbit [99]muscles when the muscle is immobilised at a shorter than optimum length and the opposite occurs when immobilised at long lengths [127, 128, 129]. In cat soleus, immobilization at short length for 4 weeks significantly reduced sarcomere number, reduced passive ROM, and increased passive muscle stiffness [121]. Subsequently, Williams and Goldspink [129] found that the loss of sarcomeres in mouse soleus after 3 weeks of immobilisation at short lengths was associated with a decreased passive ROM and increase in stiffness, and Williams et al. [131] replicated these findings despite the imposition of brief stretches during the immobilisation period. Such evidence is consistent with serial sarcomere number influencing muscle stiffness, and thus potentially ROM_max_ (Table 1).

In contrast, the significant increases in sarcomere number triggered by Tabary et al. [121] and Williams and Goldspink [129] were not associated with detectable changes in passive ROM or stiffness. Subsequently, De Jaeger et al. [31] examined the effects of intermittent cyclic stretching (3 times per week of 25 stretching cycles) during 4 weeks of immobilisation of rabbit ankle plantar flexors. Neither titin isoforms nor the total amount of collagen was altered in the small samples of the muscles tested after immobilisation with stretch training, and stiffness remained similar (from pre- to post-training) despite there being a 9.3% increase in soleus serial sarcomere number (changes were not detected in plantaris or medial gastrocnemius). While these findings suggest that serial sarcomere number can be increased by intermittent stretching during immobilisation, such (moderate) changes do not appear to substantively alter whole muscle passive stiffness. One possibility is that passive properties were strongly influenced by titin isoform (although see Sect. 2.1) or collagen content (see Sect. 2.3), which remained unchanged.

Nonetheless, it was previously concluded, based on a review of the then-available evidence, that while the magnitude of the effect of myofilaments, and thus sarcomeres, on stiffness remained unclear, changes in the constituents and orientation of connective tissues were likely key predictors of whole muscle–tendon stiffness [53, 67, 86]. On the basis of the reviewed studies, changes in extracellular connective tissues might be considered influential to whole-muscle passive stiffness after periods of stretch or immobilisation. For example, Williams et al. [131] showed that prolonged stretch of animal muscles significantly increased serial sarcomere number (~ 3000 sarcomeres, which should theoretically reduce passive stiffness), yet passive tension also increased, and ankle joint ROM decreased. Simultaneously, connective tissue content increased, which was thus suggested to underpin the observed increases in whole muscle stiffness, i.e. changes in the connective tissues were more influential than the changes in sarcomere number. Changes in connective tissue properties might also explain findings that increases in sarcomere number after cyclic, repetitive, or intermittent stretch training activities had no effect on passive stiffness [31]. These results suggest that increases in sarcomere number within normal physiological limits might not meaningfully affect whole muscle passive stiffness, at least in animal models in which substantial sarcomere number alterations have been elicited but other adaptations might also simultaneously exist. (Table 1).

The effects of changes in sarcomere number, or potential simultaneous changes in connective tissues, have not been examined in humans. Boakes et al. [18] showed that chronic stretch stimulus imposed on vastus lateralis through limb distension surgeries to lengthen the femur over 8 months triggered a significant increase in sarcomere number. However, while the resulting increase in muscle length allowed the muscle to work at the new, longer lengths imposed by the surgeries, ROM_max_ and muscle stiffness outcomes were not examined. In another recent study [2], 9 weeks of eccentric strength training of the hamstrings muscles produced a significant sarcomere number increase in biceps femoris (long head), but again no data were provided in relation to ROM_max_ or stiffness. Thus, the relationship between sarcomere number, or changes in it, and ROM_max_ and stiffness has not been directly examined in humans. Whether relatively brief bouts of stretching (e.g. several minutes per day) are sufficient to increase sarcomere number sufficiently to influence ROM_max_ or stiffness (in either healthy muscle or in clinical populations) in humans has yet to be determined.

### Muscle Fascicle Contributions

Stretch of pennate muscles does not only occur through fibre lengthening (and therefore fascicle lengthening), but also through fibre rotation or longitudinal shear at the fibre or fascicle levels. After short-term (e.g. several weeks) or longer-term (e.g. 24 weeks) muscle stretching training, no increments in resting fascicle or muscle lengths have been observed [14, 15, 37, 65, 89] a conclusion supported by recent meta-analyses [60]. Although fascicle length might be less relevant, fascicles also rotate as muscle length changes. Blazevich et al. [14] observed that fascicle rotation was greater in healthy flexible versus less flexible adults, suggesting that fascicle rotation capacity may be more important for flexibility than fascicle lengthening, especially for healthy populations with average flexibility. However, few studies have examined the role of fibre rotation or its change with training on whole muscle elongation, so more data are needed to determine their effects on passive muscle stiffness.

### Muscle Extracellular Matrix (ECM)

The muscular ECM is comprised of numerous structural proteins; however, it is commonly understood that muscle collagen content and architecture especially influence its load-deformation properties [28, 58, 135]. It is therefore of interest to examine associations between a muscle’s ECM material and mechanical properties and its passive stiffness.

Intramuscular connective tissues comprise a complex network of collagen fibres and associated matrix that is organised into three hierarchical levels: endomysium surrounding the muscle fibres, perimysium surrounding muscle fascicles, and epimysium enveloping the whole muscle [27, 28, 80] (Fig. 1). The ECM plays an important role in transferring forces produced within the muscle fibres to the tendon and ultimately to the bones [28]. Collagen is the most abundant protein within the ECM [132]. Hayat et al. [52] pointed to the ECM as a potential source of changes in passive stiffness of immobilised cat soleus muscle fibres in their study because, in muscles immobilised at short length, a ‘greater abundance of connective tissue was observed in the muscle belly of immobilized muscles when dissecting out the single fibres for the histological measurements’. However, no quantitative analysis was completed in that study. Subsequently, Williams and Goldspink [130] found that an 8% increase in perimysium content was observed alongside decreases in passive rat muscle extensibility and increased stiffness after two days of immobilisation in a shortened position, despite a lack of change in serial sarcomere number. Such results suggest that fascicle elongation is strongly affected by changes in connective tissue content, which might thus explain the decreases in whole muscle elongation. However, rapid MTU adaptations to immobilisation, including increased stiffness, have also been associated with the realignment of collagen fibres [34, 128], indicating that realignment might influence fascicle, and thus whole muscle, passive stiffness as well.

More evidence suggesting that the muscle’s ECM is a major source of the passive tension within the muscle stems from the study of Brown et al. [20], who aimed to determine the passive mechanical properties (stiffness and slack sarcomere length) of rat muscles at the fibre bundle and single fibre levels as well as the molecular mass of titin. Single rat muscle fibres and small fibre bundles were isolated and fixed to a force transducer, then lengthened until they generated passive force. Fibre bundles were assessed and compared on the descending limb of their length-tension relation and titin mass was quantified using gel electrophoresis. These investigators found that fibre bundles became stiffer than single fibres at longer whole muscle lengths, suggesting that the ECM strongly influences fibre bundle stiffness whilst titin isoform was not associated with fibre and fibre bundles stiffness. Consistent with these findings, Reyna et al. [108] decellularized avian muscles to isolate and maintain an intact ECM by removing actin and myosin from the surrounding ECM. Actin and myosin, therefore, could not influence stiffness, and titin could not function as a spring under these circumstances [106]. Using these preparations, they demonstrated that ECM content accounted for about half the overall passive stiffness in the muscle. Nonetheless, more studies are needed to examine the effects of long periods of loading (stretching) and unloading to quantify the importance of connective tissue content to temporal changes in muscle passive stiffness, since it is well documented that connective tissue realignment occurs during short periods of passive stretch or active contraction.

According to the above findings, at least according to the data from animal models, ECM appears to be an important regulator of muscle passive stiffness based on: (1) the limited influence of (moderate) sarcomere number changes and titin properties on fibre bundle and whole muscle passive stiffness, (2) the moderate capacity for the ECM to adapt (e.g. passive or active stretches) and (3) the influence of the ECM on fascicle lengthening [135]. If this is true, then alterations in the ECM might be an important target for reducing whole muscle passive stiffness, and possibly improving ROM_max_, and may explain the substantial decrease in muscle stiffness (and possibly ROM_max_) observed after interventions such as strength training at long muscle lengths or eccentric muscle contractions [16, 48, 59, 63]. It is important to note that much of our current understanding is based on animal models. However, recent studies have demonstrated the capacity of microstructures within the ECM to adapt and remodel [84]. Future research may provide additional insight into the role of the ECM function and its adaptability to changes in physical activity.

## Tendon

Classical literature has highlighted the importance of tendons during muscle contraction and other forms of movement. Tendons are also primarily composed of collagen fibre bundles and serve the muscle–tendon unit (MTU) as a protective and force-transmitting structure when the joint is under tension  [100]. In conditions where the MTU is loaded, such as during stretching exercises, the muscle can absorb part of the applied tension. Tendons are therefore adapted to accommodate high tensile loads, particularly during isometric conditions [100]. One hypothesis, therefore, is that decreases in tendon stiffness might enable a lower MTU stiffness and increase in ROM_max_.

However, while some studies have observed relationships between tendon stiffness and the resistance to stretch (e.g. at the ankle joint) [60], there is little evidence that tendon stiffness is a key influencing factor of either ROM_max_ or stiffness. For example, after an acute stretching intervention such as passive stretching, e.g. lasting 2–5 min, where the MTU is under sustained tension, ROM_max_ is increased significantly and MTU stiffness is sometimes reduced [65, 88] yet tendon mechanical properties appear to remain largely unchanged [65, 88], and in one study tendon stiffness was observed to increase [89]. Additionally, chronic stretching training has also been shown to significantly increase ROM_max_ without detectable changes in tendon stiffness (e.g. increased ankle ROM_max_ without change in Achilles tendon stiffness) [14, 22, 63], as confirmed by meta-analysis [24].

Additional evidence for the lack of influence of tendon properties on ROM_max_ or stiffness derives from studies using other exercise interventions. Chronic eccentric strength training sufficient to produce significant (31%) Achilles tendon stiffness increase and gastrocnemius medialis passive stiffness decrease (~ 15%) generated a substantial increase in ROM_max_ (~ 15°) without change in whole MTU (joint) stiffness [63] i.e. an increase in tendon stiffness could be evoked whilst ROM_max_ also improved. Additionally, Mahieu et al. [81] observed increases in ROM_max_ and decreases in stiffness (measured through a submaximal ROM) after 6 weeks of heel drop (calf muscle) exercise without detectable change in Achilles tendon stiffness, and Gomes et al. [44] found no relationship between back squat depth and Achilles tendon stiffness, collectively suggesting that ‘active’ exercises produce effects exclusive of tendon adaptations, and ROM during active tasks is not predicated on tendon stiffness. In fact, significant increases in ROM_max_ can be produced by strength training through a large ROM despite substantial increases in tendon stiffness being generated (e.g. see [24] for a review). Thus, on the basis of current evidence in human studies, both acute and chronic changes in ROM_max_ and stiffness (with less evidence for the latter) are not coupled to specific changes in tendon stiffness. In fact, it is both notable and practically meaningful that significant increases in ROM_max_ can be produced despite significant increases in tendon stiffness.

## Intramuscular Fat

Intramuscular fat (adipose tissue) can be found in intramyocellular (within muscle cells) and extramyocellular (outside of muscle cells) depots [103]. Adipose cells have different mechanical properties to the longer muscle cells (fibres) they reside amongst, and their ECM will further affect their properties [103]. Thus, intramuscular fat infiltration may be expected to influence muscle stiffness and therefore potentially influence ROM_max_. In line with this, Giambini et al. [41] tested the relationships between cadaveric (age 25–94 years, median 78 years) supraspinatus adipose content (measured using magnetic resonance imaging [MRI] scanning) and both muscle stiffness and extensibility (i.e. stretch distance under a 60-N load) and found that significantly increased adiposity was associated with a small but statistical reduction in stiffness but greater extensibility. Thus, adiposity tended to slightly reduce passive muscle stiffness, but it did not preclude the muscle from stretching to a long length.

Nonetheless, D’ Souza et al. [30] tested the relation between intramuscular fat assessed by MRI and ROM_max_ using an electronic goniometer in children with contracture secondary to cerebral palsy. Although increases in intramuscular fat were associated with increases in muscle cross-sectional area (which theoretically should decrease ROM_max_ because of the greater passive stiffness, e.g., [79]), intramuscular fat was not associated with ROM_max_, thus indicating that other factors play a greater role in the changes in stiffness and ROM_max_ in this population. However, given that many other factors influence muscle stiffness in people with cerebral palsy, including the sarcomeres resting at longer lengths and adaptations within the ECM, such data do not preclude adipose tissue from being relevant in other, especially non-clinical, populations [74, 75, 83].

More recently, Yoshiko et al. [134] tested the relation between plantar flexor passive elastic modulus estimated using shear-wave elastography and both gastrocnemius medial cross-sectional area and total adipose content using MRI. Consistent with previous data e.g.  [25] they found no relationship between adiposity and shear-wave speed (indicative of modulus) when the muscle rested at plantar flexed or neutral joint angles. However, they observed a negative correlation when testing with the ankle in 15° dorsiflexion, and thus with the muscle at a longer length, indicating an increased modulus with lower adiposity. One issue with shear-wave measurements is that they estimate the ‘state’ modulus of the tissue (i.e. the properties in a structure’s current state) and this may not indicate the true stiffness of a structure or material during ongoing deformation, i.e. stretch, because changes in ECM properties and internal friction (viscoelastic) effects will influence properties during stretch. Nonetheless, the researchers found a negative relationship also between adiposity and the change in shear-wave speed (i.e. modulus) from the neutral to dorsiflexed positions. Such data may indicate that adiposity reduces the change in modulus as muscle length changes, indicating that it affects muscle extensibility.

On the basis of current evidence, intramuscular fat might reduce passive muscle stiffness, but further studies are needed, and there is a lack of research evidence supporting its effects on ROM_max_ (Table 1). Given that adipose tissue properties are also rate-dependent, further examination of the effect of adiposity on stiffness and ROM_max_ under faster stretch speeds is also needed [39].

## Intramuscular Water

Water exists in substantial amounts in both muscle intracellular and extracellular spaces as well as in blood vessels [115]. Intramuscular fluid pressure is affected by passive elongation as well as active muscle contraction, so it affects muscle stretch under both relaxed [115] and active (with muscle contraction) stretch conditions [125]. This is because the muscle fibres, and the whole muscle, must decrease in cross-sectional area as the muscle elongates because muscles must remain (almost) isovolumetric. Thus, increases in fluid pressure within the muscle will resist the decrease in cross-sectional area and thus must increase the resistance to muscle lengthening [116]. Sleboda and Roberts [116] examined the interaction between intramuscular fluid and ECM mechanical function by comparing passive stiffness and active muscles in an animal model and gaining mechanistic insight by a series of physical models. This physical model was demonstrated utilising a helical sleeve of flexible plastic fibres, representing muscle ECM, and a water-filled bladder representing the incompressible volume of intramuscular fluid. The model was stretched by a specific level of force and repetitive stretches were performed with incrementally higher fluid volumes. The investigators found that an increase in fluid pressure decreased isometric force at short muscle lengths and increased isometric force at long muscle lengths, with no effect at intermediate lengths. Therefore, changes in ECM passive force may be linked to alterations in intramuscular water which alters its stiffness, implicating both water content and ECM properties in muscle stiffness (Table 1).

In addition to the above, the reorientation of the muscle ECM during passive stretches allowed the whole muscle to stretch; however, collagen reorientation was reduced when water pressure was higher, increasing ECM passive tension and thus stiffness [115]. Accordingly, both passive and active stiffness should increase with increasing fluid accumulation because fluid pressure affects radial compression, consistent with observations after fatiguing muscle contractions leading to increased intramuscular water content [69, 95]. Additionally, this increase in pressure might speculatively alter afferent signals back to the spinal cord and brain, which could influence the decision to continue to stretch further. This should affect ROM_max_, although this hypothesis has yet to be explicitly tested. Regardless of the exact mechanism, increases in muscle water content tend to increase passive stiffness, whilst decreases should reduce it. With ageing, for example, decreases in intracellular water content tend to occur [77, 133] which should possibly then alter passive stiffness (although note that changes in the ECM also occur). Whilst changes in fluid pressures might influence sensory feedback and the increased stiffness might affect extensibility, it is presently unclear whether, or to what magnitude, intramuscular water influences ROM_max_.

## Capsule, Ligament, and Articular Connective Tissue Effects on Joint Maximum Range of Motion and Stiffness

Joint capsules are important for the protection of synovial joints through joint stabilisation as well as the provision of proprioceptive information via their sensory innervation. Joint capsules are thick (depending on the joint) connective tissues that are attached to the bones and encapsulate the joints. There are many subdivisions of the material of the joint capsules, which consist of mainly elastic fibres, collagen, proteoglycans and glycosaminoglycans [10]. Synovial fluid responds to musculoskeletal trauma, inflammation, and disease [112] and may be reduced or inflamed by these conditions. Before corrective posttraumatic surgery and whilst under anaesthesia (meaning the muscles were fully relaxed), Gallay et al. [39] tested the capacity and compliance (the amount of intraarticular pressure) of patients’ elbow joint capsules in both the stiff and normal elbows. The researchers found that joint capsules in those with reduced ROM (posttraumatic elbow joint contractures with no history of connective tissue disorder) were 15% stiffer than the normal individuals and the mechanical aspects and the joint structures explained these differences. Stiff joints have been associated with a reduced ROM_max_, which can cause painful intra-articular pressure, and thus some studies have used methods to define the elbow joint positions at which minimum and maximum intra-articular pressures occur [39, 46]. A possible explanation for the reduced ROM_max_ might be the sensation of stiffness at the end of the ROM_max_ (as discussed below). Along with the musculoskeletal mechanical response to a stretch that increases passive stiffness, the amount of intra-articular pressure in the joint caused by inflammation, loose bodies, and synovial thickness was also associated with lesser ROM_max_ in individuals with poor ROM_max_ before and after arthroscopic surgery [46]. Whether this resulted directly from the increase in stiffness causing joint limitation or the altered sensations from the moving limb influencing the decision to stretch further is unclear. Regardless, at least in some individuals (e.g. older or those with joint conditions or disease), joint structures may influence both stiffness and ROM_max_, although in non-clinical populations they may play a lesser role. Nonetheless, further information regarding the impact of internal joint structures may arise if future studies are completed on joints other than the commonly examined elbow joint (e.g. the knee or ankle).

## Sensory Contribution to Joint Maximum Range of Motion

Additional to any mechanical influences on stiffness and ROM_max_, the sensations felt during stretch should also influence our decision to resist stretch (increasing stiffness) or stop a stretch before our maximum limit (reducing ROM_max_) [125]. Many sites along the neuromuscular pathway may therefore influence both stiffness and ROM_max_.

Muscle spindles, and more specifically the nuclear bag and chain fibres, are sensory receptors responsible for detecting the rate and magnitude of muscle length. Those sensory signals are transmitted to the spinal cord and supraspinal sensory areas, including the cerebellum. Muscle spindle signals contribute to the sensations of limb position and movement, providing conscious information regarding where we are and how we are moving in space [119]. Muscle spindles show history-dependent behaviour, which modulates their activity and affects an individual’s acuity to detect movement and afferent transmission to the spinal cord. Short durations (several minutes) of muscle or tendon vibration, which strongly stimulate muscle spindles to cause both a reduction in spindle pathway excitability [109] and the illusion of movement (altering our perception of a joint’s angle or muscle’s length) [45, 107, 120] can acutely reduce stiffness and increase ROM_max_ (for a review, see [61]). Such findings are consistent with, although not direct proof of, an effect of muscle spindle activation on flexibility.

Spindle information transmission to spinal motoneurons is commonly assessed by H-reflex measurements [49]. In an early study, Nielsen et al. [94] found that H-reflexes in the plantar flexor muscles were smaller in highly flexible ballet dancers than in untrained controls, suggesting a link between reduced excitability of the spindle pathway and increased ROM_max_. However, cross-sectional studies are problematic because of factors such as the ‘relative’ muscle length (dancers are more flexible, so they might need to be tested at a longer muscle length, i.e. common percentage of maximum muscle length), motor unit type (fast versus slow), and others, can influence reflex amplitudes [47]. Subsequently, Guissard and Duchateau [49] found decreases in both the H-reflex and the tendon tap reflex (T-reflex), representing Ia afferent feedback from muscle spindles or muscle spindle sensitivity, respectively, after 30 sessions of calf muscle stretching training. Nonetheless, the reduction in T-reflex occurred more rapidly than the reductions in H-reflex (after 10–20 sessions), which might indicate that there was a greater change in spindle sensitivity or enhanced Golgi tendon organ (GTO) response. Furthermore, muscle–tendon unit stiffness decreased along with increases in ankle ROM_max_ and changes in H and T-reflex, consistent with the hypothesis that spindle information might influence both stiffness and ROM_max_, and their temporal association is consistent with the idea that changes in information transmitted through the muscle spindles and associated pathways might influence muscle activity during stretch as well as the final ROM achieved. H-reflex and M-wave returned to baseline (pre-training) by 30 days after training cessation, suggesting further that the changes in passive stiffness might be at least partly explained by changes in reflexive activity.

Nonetheless, in a subsequent study, Blazevich et al. [15] found decreases in gastrocnemius muscle stiffness as well as soleus and gastrocnemius medial H reflex amplitudes when measured with the ankle joint in plantar flexion (i.e. at short muscle length) and the neutral position but not with the ankle in dorsiflexion, and thus with the muscle ‘on stretch’. Therefore, no change in the stretch reflex pathway was detected when measured with the muscles in the stretched position, suggesting that changes in stiffness and ROM_max_ may not be directly related to changes in muscle spindle-derived sensory information. It is also important to highlight that many studies fail to detect differences in electromyography (EMG) onset angle, maximum EMG amplitude, or EMG amplitude at stretch termination, or a change with stretching training leading to ROM increases [80], which might otherwise be expected if spindle or spindle pathway function was altered by stretch training. Additionally, Blazevich et al. [14] found a greater ankle joint ROM_max_ in “flexible” individuals than in inflexible but no differences in the H: M ratios or any EMG variables between them when tested at neutral and dorsiflexed positions (i.e. at longer muscle lengths). Finally, Anvar et al. [4] found significant increases in ankle dorsiflexion ROM_max_ in both stretched and non-stretched (control) limbs, yet changes in *H*_max_/*M*_max_ ratio were observed only in the stretched limb, suggesting that acute adaptations in spindle information transmission to spinal motoneurons may occur with stretching but are not required for significant ROM_max_ improvements. Thus, although changes in H and T reflexes can be observed after training, and differences exist between individuals, it does not appear that these meaningfully affect stiffness or ROM_max_.

Other sensory receptors, which remain to be extensively studied, also exist in the joints, skin, and ligaments. For example, similar to muscle spindles, illusions of movement have been produced by stretching the skin [13, 26, 34] or electrically stimulating the skin on the back of the hand [26, 27], suggesting that cutaneous (skin) receptors influence kinaesthetic perception and may thus influence ROM_max_ or resistance to stretch. However, their association with muscle stiffness and ROM_max_ remains to be explicitly tested. Likewise, joint ROM_max_ might be influenced by GTO activities. If increases in muscle force during stretch can activate GTOs, and if under these conditions GTO activity primarily inhibits muscle activation, then one might expect reduced reflexive stiffness and possibly greater ROM_max_ in people with more sensitive GTOs, or at least with greater GTO pathway excitability. GTO activity has, to date, not been explicitly studied, and therefore it is not yet possible to draw conclusions about its role in joint flexibility. Finally, joint receptors are sensitive to changes in pressure within the joint, which may change as a joint is rotated, and the associated muscle–tendon units exert pressure as they are strained [35]. Theoretically, this may impact the perception of the stretch, thus influencing the voluntary activation of muscles during the stretch or the decision to stop the stretch at acute angles. However, this hypothesis has yet to be explicitly tested.

Another possibility is that spinal-level adaptations, potentially modulated by supraspinal commands, might influence the array of sensory feedback across afferent pathways to contribute to altered reflexes during stretch. As evidence of this, soleus motor evoked potential (MEP) amplitudes are smaller with the ankle in dorsiflexion than in an ‘off stretch’ condition [49]. Therefore, afferent information seems to be depressed at the spinal level as the muscle lengthens, and this phenomenon appears to be enhanced in more flexible individuals or after a period of muscle stretch training [50, 93]. Furthermore, even after 5 min of plantar flexor stretches that did not produce a detectable change in T reflex amplitude or latency after tendon tap, a significant reduction in the soleus late reflex response, which is observed in the EMG after the MEP response to cortical stimulation, has been observed [98]. This depressed response is thought to indicate reduced excitability of cerebellar-derived neural pathways (or the cerebellum itself), which are central to motor pathway function and movement performance [68, 118]. Thus, complex spinal-supraspinal networks may be affected by stretching, which then modulate sensory reception and interpretation. Such networks have yet to be studied in detail.

Research evidence also suggests that increased pain tolerance may be associated with increases in ROM_max_, although less information exists regarding its impact on passive stiffness. The increased sensory information from pain receptors may be used to ensure individuals do not exceed their stretch (or joint range) limit, so pain may reduce the likelihood that individuals rotate a joint or stretch a muscle, and may also increase reflexive or voluntary muscle activity during a stretch, thus increasing resistance to stretch (stiffness) [64, 117]. For example, Stove et al. [117] found an increased knee passive torque (i.e. stiffness) in a group reporting widespread pain than a non-painful control group. This could suggest that that pain sensitivity can influence passive stiffness and ROM_max_. Stretching exercises are purported to promote hypoalgesia, reducing pain in patients with chronic pain (e.g., low back pain) [8, 64], although the stretch itself may need to be strong enough to elicit a pain response [1]. Therefore, pain sensation might be associated with changes in ROM_max_ and possibly partly explain why some clinical individuals have difficulty stretching further [96].

Nonetheless, some evidence suggests that alterations in pain sensitivity to stretch might be linked to changes in spindle-associated stretch feedback. While stretch training appears to increase pain tolerance, the mechanisms underlying improvements in ROM_max_ cannot be fully explained by changes in pain perception alone. For example, following a period of stretch training, pain sensations may become more prominent at the beginning of a stretch or during the first set [82]. Additionally, the relationship between stretching and pain perception involves competing neural mechanisms. An acute bout of stretching can induce muscle spindle sensitisation, whereby muscle spindles exhibit heightened responsiveness to lengthening [78]. The possibility exists that the elevated spindle activity may temporarily compete with, or override, nociceptive input, consistent with gate control theory [22], allowing an individual to stretch further even whilst feeling some pain. However, if repeated stretching within a session promotes muscle spindle desensitisation (described above), then afferent discharge during stretch may be reduced and thus perception of pain increased. If this is the case then any alterations in pain that influence either stiffness or ROM_max_ must result from different physiological phenomena, such as alterations in endogenous pain modulation pathways (e.g. see [8]); however, this hypothesis needs to be explicitly tested. Such neural adaptations should not only decrease reflexive resistance to lengthening but may also diminish the dominance of spindle signalling relative to pain receptors, thereby enhancing stretch tolerance and facilitating gains in ROM.

## Interpretation of Sensory Information

Sensory information, including from muscle spindles and other receptors such as cutaneous afferents and pain receptors, is ultimately transferred to the somatosensory cortex via the medial lemniscus (cutaneous), spinocerebellar (muscle length and length change) and spinothalamic (e.g. pain, pressure) pathways. The information is then processed in sensory association areas in the parietal lobe [3]. These areas essentially compare this information to what has been learned before so that the information has context. Once it has context, it can be sent to the frontal lobe for decisions to be made as to how to respond [90]. The frontal lobe helps make these decisions before the individual then enacts a plan, either to allow a stretch to continue or to endorse a motor strategy to stop the stretch, e.g. by withdrawing the limb, activating muscles to resist further stretch, or altering the situation in some other manner (such as pressing a “stop’ button in the dynamometer if the test is performed in this device in a laboratory). Therefore, if stretch tolerance is an important factor influencing ROM_max_, and this might be influenced by perceptions of sensory signals such as the amount of muscle or skin stretch or pain or pressure magnitudes, then stretch tolerance mechanisms might be explained by how sensory information is transmitted to the sensory cortex and then used for decision-making relating to stretch amplitude_._ This sensory information can be used to perceive how a joint is rotated in space, thereby improving the information to decide when to stop the stretch. One might hypothesise, then, that the interpretation of sensory information its magnitude or type, might affect how the stretch is tolerated and thus whether the stretch is resisted (increasing muscle stiffness) or stopped (reducing ROM_max_). Therefore, by altering the magnitude or type of afferent feedback or influencing the decision-making propensity in response to the sensory information, one might acutely impact either or both stiffness and ROM_max_; such an outcome would be consistent with the known effects of mental state (including anxiousness, confidence, etc.) on perceptions of other physical senses such as pain [22].

As for now, however, limited research has examined the influence of decision-making processes on either stiffness or ROM_max_. This might be done, for example, by altering mental state using either calming or arousing environments, performing motivational or relaxing mental tasks (e.g. ‘psyching up’ or performing mindfulness practices), or by altering the activity of areas of the brain associated with risk-taking decisions (e.g. frontal lobe) through mental tasks or direct cortical (e.g. transcranial) activation. One recent study used motivational versus relaxing music before participants stretched their calf muscles, and the effects appeared to be relatively small, with a reduction observed in stiffness during faster stretches (30° ankle dorsiflexion rotations) after relaxing music but no changes in ROM_max_ observed [76]. Much more work needs to be done, including after interventions that influence arousal or decision-making behaviours more than the brief playing of music. Altering the activities of decision-making areas may thus alter flexibility acutely or chronically, and this may be a fertile area for future research.

Taken together, the evidence reviewed above supports a dual-mechanism model of joint flexibility in which passive stiffness and ROM_max_ are regulated by partially dissociable processes. Passive stiffness appears to be principally a tissue-level phenomenon, governed by the mechanical properties of the ECM, intramuscular fat and water, and fascicle rotation capacity, although neural contributions, whether reflexive or voluntary (e.g. muscle activation associated with pain-induced guarding), can modulate the resistance to stretch under certain conditions. ROM_max_, in contrast, is additionally and perhaps more fundamentally shaped by neuro-perceptual and cognitive processes, specifically the interpretation of proprioceptive, nociceptive and mechanoreceptive signals within higher brain centres and the consequent decisions regarding stretch tolerance. The practical implication is that stiffness and ROM_max_ need not change in concert; indeed, significant improvements in ROM_max_ have been observed in the absence of detectable changes in passive tissue stiffness, and vice versa. A dual-mechanism model therefore provides a parsimonious framework for understanding this dissociation, and for guiding the design of interventions that may need to address tissue mechanics, neuro-perceptual processes, or both, depending on the outcome sought.

## Priorities for Future Research

Future research on joint flexibility should adopt a more integrated and human-centred approach. Current evidence highlights a significant gap in human-specific data, particularly regarding muscle architecture and its relationship to stiffness. For instance, the role of sarcomere arrangement in humans remains largely unexplored. Investigating the relationship between serial sarcomere number and both whole muscle stiffness and ROM_max_ is essential to better understand structural determinants of flexibility. In addition, further research is needed to clarify how the ECM regulates passive muscle stiffness as well as to determine the individual and combined contributions of secondary mechanical factors such as fascicle rotation capacity, intramuscular fat, and water content to overall muscle resistance.

Beyond mechanical properties, joint flexibility is strongly influenced by neuro-perceptual and cognitive mechanisms. Since ROMₘₐₓ is critically shaped by sensory perception rather than purely structural constraints, future studies should focus more on cortical and sub-cortical processing. Specifically, research should investigate how higher brain centres interpret proprioceptive input from muscle spindles, nociceptors, and joint mechanoreceptors to establish stretch tolerance. Additionally, the role of psychological states and decision-making processes in modulating voluntary ROM_max_ should be explicitly examined, independent of tissue-level properties.

Finally, future work should aim to develop integrated intervention strategies that reflect this dual-mechanism model of flexibility. This includes exploring whether cognitive-targeted interventions, such as those influencing perception or decision-making, can enhance flexibility alongside traditional mechanical approaches like stretching. Moreover, understanding the interaction between neuromechanical factors and cortical processing may help inform more effective and functionally relevant strategies for both clinical rehabilitation and performance optimisation.

## Conclusions

Joint flexibility encompasses both ROM_max_ and resistance to stretch (stiffness), with each influenced by distinct but interrelated mechanisms. Current evidence indicates that whole muscle passive stiffness is predominantly regulated by the extracellular matrix ECM stiffness (and thus its structure and organisation), with additional contributions from fascicle rotation capacity, intramuscular fat, and water content. While titin governs sarcomere-level stiffness, its influence diminishes at the whole muscle level, where ECM properties appear more determinative. Serial sarcomere number changes within physiological ranges appear to have limited impact on whole muscle stiffness in animal models, particularly when concurrent ECM adaptations occur, but relations between serial sarcomere number and stiffness or ROM_max_ have not been examined in human studies. ROM_max_, on the other hand, is influenced not only by these mechanical factors but critically by sensory‒perceptual processes. Proprioceptive information from muscle spindles, pain receptors and joint mechanoreceptors is interpreted within higher brain centres, where decisions regarding stretch tolerance are made. This cognitive component suggests that psychological state, pain perception and decision-making processes may substantially influence voluntary ROM_max_, independent of tissue mechanical properties. While research is required to explicitly interrogate these hypotheses, the evidence reviewed here is consistent with a dual-mechanism model of flexibility in which passive stiffness is principally determined at the tissue level while ROM_max_ additionally reflects neuro-perceptual decision-making. The dissociation sometimes observed between changes in stiffness and ROM_max_ following interventions is consistent with this model. Future research should investigate how cortical processing of sensory information influences stretch tolerance and whether interventions targeting decision-making processes can enhance flexibility alongside traditional mechanical approaches. Understanding these integrated neuromechanical mechanisms will inform more effective strategies for improving functional flexibility in clinical and performance contexts.

## References

[1] Anderson AW, Soncini A, Lyons K, Hanney WJ. The effect of myofascial stretching on mechanical nociception and contributing neural mechanisms. NeuroSci. 2024;5(2):158–68. 10.3390/neurosci5020011 PMC11493203 39483492

[2] Andrews MH, Pai A, Gurchiek RD, Pincheira PA, Chaudhari AS, Hodges PW, et al. Multiscale hamstring muscle adaptations following 9 weeks of eccentric training. J Sport Health Sci. 2025;14:100996. 10.1016/j.jshs.2024.100996 PMC11863337 39461588

[3] Andersen RA. The neurobiological basis of spatial cognition: role of the parietal lobe. In: Spatial cognition. Psychology Press; 2022. p. 57–80.

[4] Anvar SH, Granacher U, Konrad A, Alizadeh S, Culleton R, Edwards C, et al. Corticospinal excitability and reflex modulation in a contralateral non-stretched muscle following unilateral stretching. Eur J Appl Physiol. 2023;123(8):1837–50. 10.1007/s00421-023-05200-9 37072505

[5] Ackerley R, Samain-Aupic L, Ribot-Ciscar E. Passive proprioceptive training alters the sensitivity of muscle spindles to imposed movements. eNeuro. 2022. 10.1523/ENEURO.0249-21.2021. PMC8805769 35022185

[6] Baethge C, Goldbeck-Wood S, Mertens S. SANRA—a scale for the quality assessment of narrative review articles. Res Integr Peer Rev. 2019;4(1):5. 10.1186/s41073-019-0064-8 PMC6434870 30962953

[7] Bartoo ML, Linke WA, Pollack GH. Basis of passive tension and stiffness in isolated rabbit myofibrils. Am J Physiol Cell Physiol. 1997;273(1):C266–76. 10.1152/ajpcell.1997.273.1.C266 9252465

[8] Behm DG, Kay AD, Trajano GS, Alizadeh S, Blazevich AJ. Effects of acute and chronic stretching on pain control. J Clin Exerc Physiol. 2021;10(4):150–9.

[9] Behm DG. The science and physiology of flexibility and stretching: implications and applications in sport performance and health. Routledge; 2024.

[10] Benjamin M, Ralphs J. Fibrocartilage in tendons and ligaments—an adaptation to compressive load. J Anat. 1998;193(4):481–94. 10.1046/j.1469-7580.1998.19340481.x PMC1467873 10029181

[11] Bielajew BJ, Hu JC, Athanasiou KA. Collagen: quantification, biomechanics and role of minor subtypes in cartilage. Nat Rev Mater. 2020;5(10):730–47. 10.1038/s41578-020-0213-1 PMC8114887 33996147

[12] Bird ML, Hill K, Ball M, Williams AD. Effects of resistance- and flexibility-exercise interventions on balance and related measures in older adults. J Aging Phys Act. 2009;17(4):444–54. 10.1123/japa.17.4.444 19940323

[13] Blanchard C, Roll R, Roll JP, Kavounoudias A. Differential contributions of vision, touch and muscle proprioception to the coding of hand movements. PLoS ONE. 2013;8:e62475. 10.1371/journal.pone.0062475 PMC3633880 23626826

[14] Blazevich AJ, Cannavan D, Waugh CM, Fath F, Miller SC, Kay AD. Neuromuscular factors influencing the maximum stretch limit of the human plantar flexors. J Appl Physiol. 2012;113(9):1446–55. 10.1152/japplphysiol.00882.2012 22923509

[15] Blazevich AJ, Cannavan D, Waugh CM, Miller SC, Thorlund JB, Aagaard P, et al. Range of motion, neuromechanical, and architectural adaptations to plantar flexor stretch training in humans. J Appl Physiol. 2014;117(5):452–62. 10.1152/japplphysiol.00204.2014 24947023

[16] Blazevich AJ. Adaptations in the passive mechanical properties of skeletal muscle to altered patterns of use. J Appl Physiol. 2019;126(5):1483–91. 10.1152/japplphysiol.00700.2018 30412028

[17] Booth FW. Effect of limb immobilization on skeletal muscle. J Appl Physiol. 1982;52(5):1113–8. 10.1152/jappl.1982.52.5.1113 7047468

[18] Boakes JL, Foran J, Ward SR, Lieber RL. Case report: muscle adaptation by serial sarcomere addition 1 year after femoral lengthening. Clin Orthop Relat Res. 2007;456:250–3. 10.1097/01.blo.0000246563.58091.af 17065842

[19] Brodie TG. The extensibility of muscle. J Anat Physiol. 1895;29(Pt 3):367. PMC1328411 17232140

[20] Brown SH, Carr JA, Ward SR, Lieber RL. Passive mechanical properties of rat abdominal wall muscles suggest an important role of the extracellular connective tissue matrix. J Orthop Res. 2012;30(8):1321–6. 10.1002/jor.22068 PMC3337947 22267257

[21] Brynnel A, Hernandez Y, Kiss B, Lindqvist J, Adler M, Kolb J, et al. Downsizing the molecular spring of the giant protein titin reveals that skeletal muscle titin determines passive stiffness and drives longitudinal hypertrophy. Elife. 2018;7:e40532. 10.7554/eLife.40532 PMC6300359 30565562

[22] Cimpean A, David D. The mechanisms of pain tolerance and pain-related anxiety in acute pain. Health Psychol Open. 2019;6(2):2055102919865161. 10.1177/2055102919865161 PMC6886278 31827877

[23] Cini A, Sonda FC, da Rocha ES, Borges M, Felappi CJ, Vaz MA, et al. Effects of passive static stretching on the Achilles tendon properties of adults: a randomized controlled trial. Sport Sci Health. 2024;20(2):337–46.

[24] Cini A, Sonda FC, de Oliveira Borges M, Vaz MA, Lima CS. Effect of acute static stretching on the passive mechanical properties of the Achilles tendon: a systematic review and meta-analysis. Int J Ther Rehabil. 2021;28(11):1–10.

[25] Chino K, Takahashi H. Measurement of gastrocnemius muscle elasticity by shear wave elastography: association with passive ankle joint stiffness and sex differences. Eur J Appl Physiol. 2016;116(4):823–30. 10.1007/s00421-016-3339-5 26874517

[26] Collins DF, Prochazka A. Movement illusions evoked by ensemble cutaneous input from the dorsum of the human hand. J Physiol. 1996;496:857–71. 10.1113/jphysiol.1996.sp021733 PMC1160870 8930850

[27] Collins DF, Refshauge KM, Gandevia SC. Sensory integration in the perception of movements at the human metacarpophalangeal joint. J Physiol. 2000;529:505–15. 10.1111/j.1469-7793.2000.00505.x PMC2270207 11101658

[28] Csapo R, Gumpenberger M, Wessner B. Skeletal muscle extracellular matrix–what do we know about its composition, regulation, and physiological roles? A narrative review. Front Physiol. 2020;11:253. 10.3389/fphys.2020.00253 PMC7096581 32265741

[29] Danesini PC, Heim M, Tomalka A, Siebert T, Ates F. Endomysium determines active and passive force production in muscle fibers. J Biomech. 2024;168:112134. 10.1016/j.jbiomech.2024.112134 38723428

[30] D’Souza A, Bolsterlee B, Lancaster A, Herbert RD. Intramuscular fat in children with unilateral cerebral palsy. Clin Biomech. 2020;80:105183. 10.1016/j.clinbiomech.2020.105183 33096341

[31] De Jaeger D, Joumaa V, Herzog W. Intermittent stretch training of rabbit plantarflexor muscles increases soleus mass and serial sarcomere number. J Appl Physiol. 2015;118(12):1467–73. 10.1152/japplphysiol.00515.2014 26078433

[32] DeLeon-Pennell KY, Barker TH, Lindsey ML. Fibroblasts: the arbiters of extracellular matrix remodeling. Matrix Biol. 2020;91:1–7. 10.1016/j.matbio.2020.05.006 PMC7434687 32504772

[33] Edin BB, Vallbo AB. Stretch sensitization of human muscle spindles. J Physiol. 1988;400(1):101–11. 10.1113/jphysiol.1988.sp017113 PMC1191800 2971105

[34] Edin BB, Johansson N. Skin strain patterns provide kinaesthetic information to the human central nervous system. J Physiol. 1995;487:243–51. 10.1113/jphysiol.1995.sp020875 PMC1156613 7473253

[35] Ferrell WR, Gandevia SC, McCloskey DI. The role of joint receptors in human kinaesthesia when intramuscular receptors cannot contribute. J Physiol. 1987;386:63–71. 10.1113/jphysiol.1987.sp016522 PMC1192450 3681720

[36] Forcinito M, Epstein M, Herzog W. A numerical study of the stiffness of a sarcomere. J Electromyogr Kinesiol. 1998;8(2):133–8. 10.1016/s1050-6411(97)00030-8 9680954

[37] Freitas SR, Mendes B, Le Sant G, Andrade RJ, Nordez A, Milanovic Z. Can chronic stretching change the muscle-tendon mechanical properties? A review. Scand J Med Sci Sports. 2018;28(3):794–806. 10.1111/sms.12957 28801950

[38] Gajdosik RL. Passive extensibility of skeletal muscle: review of the literature with clinical implications. Clin Biomech. 2001;16(2):87–101. 10.1016/s0268-0033(00)00061-9 11222927

[39] Gallay SH, Richards RR, O’Driscoll SW. Intraarticular capacity and compliance of stiff and normal elbows. Arthroscopy. 1993;9(1):9–13. 10.1016/s0749-8063(05)80336-6 8442838

[40] Gefen A, Haberman E. Viscoelastic properties of ovine adipose tissue covering the gluteus muscles. J Biomech Eng. 2007;129(6):924–30. 10.1115/1.2800830 18067398

[41] Giambini H, Hatta T, Rezaei A, An KN. Extensibility of the supraspinatus muscle can be predicted by combining shear wave elastography and MRI-measured quantitative metrics of stiffness and volumetric fat infiltration: a cadaveric study. Clin Biomech. 2018;57:144–9. 10.1016/j.clinbiomech.2018.07.001 PMC6052767 29986277

[42] Gillies AR, Chapman MA, Bushong EA, Deerinck TJ, Ellisman MH, Lieber RL. High resolution three‐dimensional reconstruction of fibrotic skeletal muscle extracellular matrix. J Physiol. 2017;595(4):1159–71. 10.1113/JP273376 PMC5309386 27859324

[43] Goldstein MA, Michael LH, Schroeter JP, Sass RL. Z band dynamics as a function of sarcomere length and the contractile state of muscle. FASEB J. 1987;1(2):133–42. 10.1096/fasebj.1.2.3609610 3609610

[44] Gomes J, Neto T, Vaz JR, Schoenfeld BJ, Freitas SR. Is there a relationship between back squat depth, ankle flexibility, and Achilles tendon stiffness? Sports Biomech. 2022;21(7):782–95. 10.1080/14763141.2019.1690569 32022631

[45] Goodwin GM, McCloskey DI, Matthews PBC. The contribution of muscle afferents to kinaesthesia shown by vibration-induced illusions of movement and by the effects of paralysing joint afferents. Brain. 1972;95(4):705–48. 10.1093/brain/95.4.705 4265060

[46] Griesser MJ, Harris JD, Likes RL, Jones GL. Synovial chondromatosis of the elbow causing a mechanical block to range of motion: a case report and review of the literature. Am J Orthop. 2011;40(5):253–6. 21734934

[47] Grosprêtre S, Martin A. H reflex and spinal excitability: methodological considerations. J Neurophysiol. 2012;107(6):1649–54. 10.1152/jn.00611.2011 22190624

[48] Gumpenberger M, Wessner B, Graf A, Narici MV, Fink C, Braun S, et al. Remodeling the skeletal muscle extracellular matrix in older age—effects of acute exercise stimuli on gene expression. Int J Mol Sci. 2020;21(19):7089. 10.3390/ijms21197089 PMC7583913 32992998

[49] Guissard N, Duchateau J, Hainaut K. Mechanisms of decreased motoneurone excitation during passive muscle stretching. Exp Brain Res. 2001;137(2):163–269. 10.1007/s002210000648 11315544

[50] Guissard N, Duchateau J. Effect of static stretch training on neural and mechanical properties of the human plantar-flexor muscles. Muscle Nerve. 2004;29(2):248–55. 10.1002/mus.10549 14755490

[51] Harbison S, Melton BF, Hunt N, Henderson N, Adams B, Westrick R. The relationship between physical mobility and firefighter occupational task performance. Int J Exerc Sci. 2023;16(3):1216. 10.70252/NUYZ9300 PMC10824302 38287970

[52] Hayat A, Tardieu C, Tabary JC, Tabary C. Effects of denervation on the reduction of sarcomere number in cat soleus muscle immobilized in shortened position during seven days. J Physiol. 1978;74(6):563–7. 745138

[53] Herbert R. The passive mechanical properties of muscle and their adaptations to altered patterns of use. Aust J Physiother. 1988;34(3):141–9. 10.1016/S0004-9514(14)60606-1 25026068

[54] Heslinga JW, Rozendal RH, Huijing PA. Unilateral immobilization affects contralateral rat gastrocnemius muscle architecture. Cells Tissues Organs. 1992;143(3):231–5. 10.1159/000147253 1632189

[55] Herzog W. Skeletal muscle mechanics: from mechanisms to function. Wiley; 2000.

[56] Herzog JA, Leonard TR, Jinha A, Herzog W. Are titin properties reflected in single myofibrils? J Biomech. 2012;45(11):1893–9. 10.1016/j.jbiomech.2012.05.021 22677335

[57] Herzog W. The role of titin in eccentric muscle contraction. J Exp Biol. 2014;217(16):2825–33. 10.1242/jeb.099127 25122914

[58] Hinds S, Bian W, Dennis RG, Bursac N. The role of extracellular matrix composition in structure and function of bioengineered skeletal muscle. Biomaterials. 2011;32(14):3575–83. 10.1016/j.biomaterials.2011.01.062 PMC3057410 21324402

[59] Hyldahl RD, Nelson B, Xin L, Welling T, Groscost L, Hubal MJ, et al. Extracellular matrix remodeling and its contribution to protective adaptation following lengthening contractions in human muscle. FASEB J. 2015;29(7):2894–904. 10.1096/fj.14-266668 25808538

[60] Ingram LA, Tomkinson GR, d’Unienville NM, Gower B, Gleadhill S, Boyle T, et al. Mechanisms underlying range of motion improvements following acute and chronic static stretching: a systematic review, meta-analysis and multivariate meta-regression. Sports Med. 2025;55(6):1449–66. 10.1007/s40279-025-02204-7 PMC12152101 40180774

[61] Jochum D, Konrad A, Lohmann LH, Cochrane D, Rittweger J, Vogel V, et al. The merit of superimposed vibration for flexibility and passive stiffness: a systematic review with multilevel meta-analysis. J Sport Health Sci. 2025;14:101033. 10.1016/j.jshs.2025.101033 PMC12137185 40021055

[62] Kawakami Y, Kanehisa H, Fukunaga T. The relationship between passive ankle plantar flexion joint torque and gastrocnemius muscle and achilles tendon stiffness: implications for flexibility. J Orthop Sports Phys Ther. 2008;38(5):269–76. 10.2519/jospt.2008.2632 18448880

[63] Kay AD, Baxter B, Hill M, Blazevich A. Effects of eccentric resistance training on lower-limb passive joint range of motion: a systematic review and meta-analysis. Med Sci Sports Exerc. 2023;55(4):710–21. 10.1249/MSS.0000000000003085 36730587

[64] Khalil TM, Asfour SS, Martinez LM, Waly SM, Rosomoff RS, Rosomoff HL. Stretching in the rehabilitation of low-back pain patients. Spine. 1992;17(3):311–7. 10.1097/00007632-199203000-00012 1533060

[65] Konrad A, Tilp M. Increased range of motion after static stretching is not due to changes in muscle and tendon structures. Clin Biomech. 2014;29(6):636–42. 10.1016/j.clinbiomech.2014.04.013 24856792

[66] Konrad A, Stafilidis S, Tilp M. Effects of acute static, ballistic, and PNF stretching exercise on the muscle and tendon tissue properties. Scand J Med Sci Sports. 2017;27(10):1070–80. 10.1111/sms.12725 PMC5479471 27367916

[67] Kovanen V, Suominen H, Heikkinen E. Mechanical properties of fast and slow skeletal muscle with special reference to collagen and endurance training. J Biomech. 1984;17(10):725–35. 10.1016/0021-9290(84)90103-9 6241196

[68] Kurokawa- Kuroda T, Ogata K, Suga R, Goto Y, Taniwaki T, Kira J, et al. Altered soleus responses to magnetic stimulation in pure cerebellar ataxia. Clin Neurophysiol. 2007;118:1198–203. 10.1016/j.clinph.2007.03.006 17452005

[69] Lall PS, Alsubiheen AM, Aldaihan MM, Lee H. Differences in medial and lateral gastrocnemius stiffness after exercise-induced muscle fatigue. Int J Environ Res Public Health. 2022;19(21):13891. 10.3390/ijerph192113891 PMC9656849 36360770

[70] Leonard TR, Herzog W. Regulation of muscle force in the absence of actin-myosin-based cross-bridge interaction. Am J Physiol Cell Physiol. 2010;299(1):C14–20. 10.1152/ajpcell.00049.2010 20357181

[71] Lieber R, Loren GJ, Friden J. In vivo measurement of human wrist extensor muscle sarcomere length changes. J Neurophysiol. 1994;71(3):874–81. 10.1152/jn.1994.71.3.874 8201427

[72] Lieber RL. Skeletal muscle structure, function, and plasticity. Lippincott Williams & Wilkins; 2002.

[73] Lieber RL, Roberts TJ, Blemker SS, Lee SS, Herzog W. Skeletal muscle mechanics, energetics and plasticity. J Neuroeng Rehabil. 2017;14(1):108. 10.1186/s12984-017-0318-y PMC5651624 29058612

[74] Lieber RL, Fridén J. Muscle contracture and passive mechanics in cerebral palsy. J Appl Physiol. 2019;126(5):1492–501. 10.1152/japplphysiol.00278.2018 PMC6589815 30571285

[75] Lieber RL, Theologis T. Muscle‐tendon unit in children with cerebral palsy. Dev Med Child Neurol. 2021;63(8):908–13. 10.1111/dmcn.14807 33426691

[76] Lima CDPD, Ruas CV, Blazevich AJ. Influence of stretch speed and arousal state on passive ankle joint mechanics. Scand J Med Sci Sports. 2024;34(12):e14774. 10.1111/sms.14774 39639795

[77] Lorenzo I, Serra-Prat M, Yébenes JC. The role of water homeostasis in muscle function and frailty: a review. Nutrients. 2019;11(8):1857. 10.3390/nu11081857 PMC6723611 31405072

[78] Macefield VG, Knellwolf TP. Functional properties of human muscle spindles. J Neurophysiol. 2018;120(2):452–67. 10.1152/jn.00071.2018 29668385

[79] Magnusson SP, Simonsen EB, Aagaard P, Gleim GW, McHugh MP, Kjaer M. Viscoelastic response to repeated static stretching in the human hamstring muscle. Scand J Med Sci Sports. 1995;5(6):342–7. 10.1111/j.1600-0838.1995.tb00056.x 8775718

[80] Magnusson SP, Simonsen EB, Aagaard P, Boesen J, Johannsen F, Kjaer M. Determinants of musculoskeletal flexibility: viscoelastic properties, cross‐sectional area, EMG and stretch tolerance. Scand J Med Sci Sports. 1997;7(4):195–202. 10.1111/j.1600-0838.1997.tb00139.x 9241023

[81] Mahieu NN, Cools A, De Wilde B, Boon M, Witvrouw E. Effect of proprioceptive neuromuscular facilitation stretching on the plantar flexor muscle‐tendon tissue properties. Scand J Med Sci Sports. 2009;19(4):553–60. 10.1111/j.1600-0838.2008.00815.x 18627559

[82] Mansoori SS, Moustafa IM, Ahbouch A, Harrison DE. Optimal duration of stretching exercise in patients with chronic myofascial pain syndrome: a randomized controlled trial. J Rehabil Med. 2020;53(1):2741. 10.2340/16501977-2781 PMC8772376 33367925

[83] Mathewson MA, Lieber RL. Pathophysiology of muscle contractures in cerebral palsy. Phys Med Rehabil Clin N Am. 2015;26(1):57–71. 10.1016/j.pmr.2014.09.005 PMC4258234 25479779

[84] Mavropalias G, Boppart M, Usher KM, Grounds MD, Nosaka K, Blazevich AJ. Exercise builds the scaffold of life: muscle extracellular matrix biomarker responses to physical activity, inactivity, and aging. Biol Rev. 2023;98(2):481–519. 10.1111/brv.12916 36412213

[85] Mayer RF, Burke RE, Toop J, Hodgson JA, Kanda K, Walmsley B. The effect of long-term immobilization on the motor unit population of the cat medial gastrocnemius muscle. Neuroscience. 1981;6(4):725–39. 10.1016/0306-4522(81)90156-1 7242912

[86] Mayne R, Sanderson RD. The extracellular matrix of skeletal muscle. Coll Relat Res. 1985;5(5):449–68. 10.1016/s0174-173x(85)80032-7 3910337

[87] Melzack R, Wall PD. Pain mechanisms: a new theory. Surv Anesthesiol. 1967;11(2):89–90.

[88] Meshel AS, Wei Q, Adelstein RS, Sheetz MP. Basic mechanism of three-dimensional collagen fibre transport by fibroblasts. Nat Cell Biol. 2005;7(2):157–64. 10.1038/ncb1216 15654332

[89] Moltubakk MM, Villars FO, Magulas MM, Magnusson SP, Seynnes OR, Bojsen-Møller J. Altered triceps surae muscle-tendon unit properties after 6 months of static stretching. Med Sci Sports Exerc. 2021;53(9):1975–86. 10.1249/MSS.0000000000002671 34398062

[90] Morelli M, Casagrande M, Forte G. Decision making: a theoretical review. Integr Psych Behav. 2022;56(3):609–29. 10.1007/s12124-021-09669-x 34780011

[91] Morse CI, Degens H, Seynnes OR, Maganaris CN, Jones DA. The acute effect of stretching on the passive stiffness of the human gastrocnemius muscle tendon unit. J Physiol. 2008;586(1):97–106. 10.1113/jphysiol.2007.140434 PMC2375574 17884924

[92] Nakamura M, Ikezoe T, Takeno Y, Ichihashi N. Acute and prolonged effect of static stretching on the passive stiffness of the human gastrocnemius muscle tendon unit in vivo. J Orthop Res. 2011;29(11):1759–63. 10.1002/jor.21445 21520263

[93] Nielsen J, Crone C, Hultborn H. H-reflexes are smaller in dancers from The Royal Danish Ballet than in well-trained athletes. Eur J Appl Physiol Occup Physiol. 1993;66(2):116–21. 10.1007/BF01427051 8472692

[94] Nielsen CS, Stubhaug A, Price DD, Vassend O, Czajkowski N, Harris JR. Individual differences in pain sensitivity: genetic and environmental contributions. Pain. 2008;136(1–2):21–9. 10.1016/j.pain.2007.06.008 17692462

[95] Nosaka K, Sakamoto K, Clarkson PM. Eccentric exercise-induced muscle damage is dependent on muscle length. Med Sci Sports Exerc. 1996;28(5):113.

[96] Padua R, Ceccarelli E, Bondi R, Campi A, Padua L. Range of motion correlates with patient perception of TKA outcome. Clin Orthop Relat Res. 2007;460:174–7. 10.1097/BLO.0b013e318046ccb7 17414170

[97] Pinto MD, Wilson CJ, Kay AD, Blazevich AJ. Reliability of isokinetic tests of velocity- and contraction intensity-dependent plantar flexor mechanical properties. Scand J Med Sci Sports. 2021;31(5):1009–25. 10.1111/sms.13920 PMC8251531 33453060

[98] Pulverenti TS, Trajano GS, Kirk BJ, Bochkezanian V, Blazevich AJ. Plantar flexor muscle stretching depresses the soleus late response but not tendon tap reflexes. Eur J Neurosci. 2021;53(9):3185–98. 10.1111/ejn.15178 33675055

[99] Pontén E, Fridén J. Immobilization of the rabbit tibialis anterior muscle in a lengthened position causes addition of sarcomeres in series and extra-cellular matrix proliferation. J Biomech. 2008;41(8):1801–4. 10.1016/j.jbiomech.2008.03.006 18460410

[100] Proske U, Morgan DL. Tendon stiffness: methods of measurement and significance for the control of movement. A review. J Biomech. 1987;20(1):75–82. 10.1016/0021-9290(87)90269-7 3558432

[101] Proske U, Panzirsch M, Weber BM. Gravity, thixotropy and human position sense. J Physiol. 2025;603:5267–71. 10.1113/JP289524 40784674

[102] Purslow PP. The structure and role of intramuscular connective tissue in muscle function. Front Physiol. 2020;11:495. 10.3389/fphys.2020.00495 PMC7248366 32508678

[103] Rahemi H, Nigam N, Wakeling JM. The effect of intramuscular fat on skeletal muscle mechanics: implications for the elderly and obese. J R Soc Interface. 2015;12(109):20150365. 10.1098/rsif.2015.0365 PMC4535407 26156300

[104] Rassier DE, Herzog W, Pollack GH. Dynamics of individual sarcomeres during and after stretch in activated single myofibrils. Proc R Soc Lond B Biol Sci. 2003;270(1525):1735–40. 10.1098/rspb.2003.2418 PMC1691424 12965002

[105] Rassier DE. Sarcomere mechanics in striated muscles: from molecules to sarcomeres to cells. Am J Physiol Cell Physiol. 2017;313(2):C134–45. 10.1152/ajpcell.00050.2017 28539306

[106] Ranatunga KW. Endothermic force generation in fast and slow mammalian (rabbit) muscle fibers. Biophys J. 1996;71(4):1905–13. 10.1016/S0006-3495(96)79389-X PMC1233657 8889165

[107] Reschechtko S, Cuadra C, Latash ML. Force illusions and drifts observed during muscle vibration. J Neurophysiol. 2018;119(1):326–36. 10.1152/jn.00563.2017 PMC5866473 28978768

[108] Reyna WE, Pichika R, Ludvig D, Perreault EJ. Efficiency of skeletal muscle decellularization methods and their effects on the extracellular matrix. J Biomech. 2020;110:109961. 10.1016/j.jbiomech.2020.109961 PMC7487037 32827769

[109] Ribot-Ciscar E, Rossi-Durand C, Roll JP. Muscle spindle activity following muscle tendon vibration in man. Neurosci Lett. 1998;258(3):147–50. 10.1016/s0304-3940(98)00732-0 9885952

[110] Salse-Batán J, González-Devesa D, Dunabeitia I, Bidaurrazaga-Letona I, Ayán-Pérez C, Sanchez-Lastra MA. Effects of stretching exercise on walking performance and balance in older adults: a systematic review and meta-analysis. Geriatr Nurs. 2025;61:479–90. 10.1016/j.gerinurse.2024.12.018 39733629

[111] Stanziano DC, Roos BA, Perry AC, Lai S, Signorile JF. The effects of an active-assisted stretching program on functional performance in elderly persons: a pilot study. Clin Interv Aging. 2009;4(1):115–20. 10.2147/cia.s4152 PMC2685233 19503774

[112] Seidman AJ, Limaiem F. Synovial fluid analysis. StatPearls Publishing; 2019. 30725799

[113] Simons DG, Mense S. Understanding and measurement of muscle tone as related to clinical muscle pain. Pain. 1998;75(1):1–17. 10.1016/S0304-3959(97)00102-4 9539669

[114] Simpson C, Kim B, Bourcet M, Jones G, Jakobi J. Stretch training induces unequal adaptation in muscle fascicles and thickness in medial and lateral gastrocnemii. Scand J Med Sci Sports. 2017;27(12):1597–604. 10.1111/sms.12822 28138986

[115] Sleboda DA, Roberts TJ. Incompressible fluid plays a mechanical role in the development of passive muscle tension. Biol Lett. 2017;13(1):20160630. 10.1098/rsbl.2016.0630 PMC5310577 28123108

[116] Sleboda DA, Wold ES, Roberts TJ. Passive muscle tension increases in proportion to intramuscular fluid volume. J Exp Biol. 2019;222(21):jeb209668. 10.1242/jeb.209668 PMC6857584 31558592

[117] Støve MP, Thomsen JL, Magnusson SP, Riis A. The effect of six-week regular stretching exercises on regional and distant pain sensitivity: an experimental longitudinal study on healthy adults. BMC Sports Sci Med Rehabil. 2024;16(1):202. 10.1186/s13102-024-00995-2 PMC11437648 39334218

[118] Suga R, Tobimatsu S, Taniwaki T, Kira J, Kato M. The soleus late response elicited by transcranial magnetic stimulation reflects agonist- antagonist postural adjustment in the lower limbs. Clin Neurophysiol. 2001;112:2300–11. 10.1016/s1388-2457(01)00689-7 11738203

[119] Taylor JL, Amann M, Duchateau J, Meeusen R, Rice CL. Neural contributions to muscle fatigue: from the brain to the muscle and back again. Med Sci Sports Exerc. 2016;48(11):2294–306. 10.1249/MSS.0000000000000923 PMC5033663 27003703

[120] Taylor MW, Taylor JL, Seizova-Cajic T. Muscle vibration-induced illusions: review of contributing factors, taxonomy of illusions and user’s guide. Multisens Res. 2017;30(1):25–63.

[121] Tabary JC, Tabary C, Tardieu C, Tardieu G, Goldspink G. Physiological and structural changes in the cat’s soleus muscle due to immobilization at different lengths by plaster casts. J Physiol. 1972;224(1):231–44. 10.1113/jphysiol.1972.sp009891 PMC1331536 5039983

[122] Ter Keurs HE, Iwazumi T, Pollack GH. The sarcomere length-tension relation in skeletal muscle. J Gen Physiol. 1978;72(4):565–92. 10.1085/jgp.72.4.565 PMC2228549 309929

[123] Thomas JT, Ayad S, Grant ME. Cartilage collagens: strategies for the study of their organisation and expression in the extracellular matrix. Ann Rheum Dis. 1994;53(8):488. 10.1136/ard.53.8.488 PMC1005385 7944631

[124] Trinick J, Tskhovrebova L. Titin: a molecular control freak. Trends Cell Biol. 1999;9(10):377–80. 10.1016/s0962-8924(99)01641-4 10481174

[125] Weppler CH, Magnusson SP. Increasing muscle extensibility: a matter of increasing length or modifying sensation. Phys Ther. 2010;90(3):438–49. 10.2522/ptj.20090012 20075147

[126] Whitehead NP, Weerakkody NS, Gregory JE, Morgan DL, Proske U. Changes in passive tension of muscle in humans and animals after eccentric exercise. J Physiol. 2001;533(2):593–604. 10.1111/j.1469-7793.2001.0593a.x PMC2278643 11389215

[127] Williams PE, Goldspink G. The effect of immobilization on the longitudinal growth of striated muscle fibres. J Anat. 1973;116(Pt 1):45. PMC1271549 4798240

[128] Williams PE, Goldspink G. The effect of denervation and dystrophy on the adaptation of sarcomere number to the functional length of the muscle in young and adult mice. J Anat. 1976;122(Pt 2):455. PMC1231915 1002614

[129] Williams PE, Goldspink G. Changes in sarcomere length and physiological properties in immobilized muscle. J Anat. 1978;127(Pt 3):459. PMC1235732 744744

[130] Williams P, Goldspink G. Connective tissue changes in immobilised muscle. J Anat. 1984;138(Pt 2):343. PMC1164074 6715254

[131] Williams PE. Effect of intermittent stretch on immobilised muscle. Ann Rheum Dis. 1988;47(12):1014–6. 10.1136/ard.47.12.1014 PMC1003657 3207382

[132] Xue M, Jackson CJ. Extracellular matrix reorganization during wound healing and its impact on abnormal scarring. Adv Wound Care. 2015;4(3):119–36. 10.1089/wound.2013.0485 PMC4352699 25785236

[133] Yamada A, Tamori M, Iketani T, Oiwa K, Motokawa T. A novel stiffening factor inducing the stiffest state of holothurian catch connective tissue. J Exp Biol. 2010;213(20):3416–22. 10.1242/jeb.044149 20889821

[134] Yoshiko A, Ando R, Akima H. Passive muscle stiffness is correlated with the intramuscular adipose tissue in young individuals. Eur J Appl Physiol. 2023;123(5):1081–90. 10.1007/s00421-023-05137-z 36637509

[135] Zhang W, Liu Y, Zhang H. Extracellular matrix: an important regulator of cell functions and skeletal muscle development. Cell Biosci. 2021;11:1–13. 10.1186/s13578-021-00579-4 PMC8011170 33789727

